# Adherence to standards of quality HIV/AIDS care and antiretroviral therapy in the West Nile Region of Uganda

**DOI:** 10.1186/s12913-014-0521-5

**Published:** 2014-11-18

**Authors:** Aldomoro Burua, Fred Nuwaha, Peter Waiswa

**Affiliations:** Makerere University School of Public Health, College of Health Sciences, Kampala, Uganda; Division of Global Health, Karolinska Institutet, Solnavägen 1, 171 77 Solna, Sweden; Management Sciences for Health, Kampala, Uganda

## Abstract

**Background:**

Over one million people in Uganda are estimated to be infected with HIV and about 20% of these were already accessing antiretroviral therapy (ART), by 2010. There is a dearth of data on adherence to antiretroviral therapy and yet high client load on a weak and resource constrained health system impacts on provision of quality HIV/AIDS care. We assessed adherence to standards of HIV care among health workers in the West Nile Region of Uganda.

**Methods:**

We conducted a cross sectional study in nine health facilities. Records of a cohort of 270 HIV clients that enrolled on ART 12 months prior were assessed. The performance of each health facility on the different indicators of standards of HIV/AIDS care was determined and compared with the recommended national guidelines.

**Results:**

We found that 94% of HIV clients at all the facilities were assessed for ART eligibility using WHO clinical staging while only two thirds (64.8%) were assessed using CD4. Only 42% and 37% of HIV clients at district hospitals and health centers respectively, received basic laboratory work up prior to ART initiation and about a half (46.7%) of HIV clients at these facilities received the alternative standard 1st line antiretroviral (ARV) regimen. Standards of ART adherence and tuberculosis assessment declined from over 70% to less than 50% and from over 90% to less than 70% respectively, during follow up visits with performance being poorer at the higher level regional referral facility compared to the lower level facilities.

**Conclusions:**

Adherence to standards of HIV/AIDS care at facilities was inadequate. Performance was better at the start of ART but declined during the follow up period. Higher level facilities were more likely to adhere to standards like CD4 monitoring and maintaining HIV clients on standard ARV regimen. Efforts geared towards strengthening the health system, including support supervision and provision of care guidelines and job aides are needed, especially for lower level facilities.

## Background

Over 34 million people worldwide, are estimated to be living with the HIV infection and 2.7 million new HIV infections as well as 1.8 million AIDS deaths occurred by the end of 2010 [1]. The devastating nature of the epidemic is likely to over burden the health systems in many developing countries, especially those in Sub-Saharan Africa which accounts for 67% of all people living with HIV and 75% of AIDS deaths worldwide [1]. HIV/AIDS disease remains a serious public health challenge in Uganda, contributing to a disproportionately high burden of morbidity and mortality. The Uganda Ministry of Health (MOH) estimated 110,000 new HIV infections to have occurred in 2008 and that the disease has killed approximately one million people, substantially lowering the life expectancy and resulting in over a million orphaned children [2].

Availability of antiretroviral drugs has contributed to global reductions in AIDS-related deaths in recent years. It is estimated that access to antiretroviral therapy (ART) in low and middle income countries increased to 6.65 million in 2010, representing 47% coverage of people eligible for treatment [1]. The provision of quality HIV/AIDS care and antiretroviral therapy is important for better health outcomes for people living with the disease. However, to be effective, provision of ART must be of the highest quality. Quality HIV/AIDS care is defined as performance according to recommended standards of care and involves interventions that are known to be safe, affordable and have ability to mitigate morbidity and mortality among HIV infected clients [3]. Standards of quality HIV/AIDS care are expectations of performance and represent a generic statement of what is expected of the HIV therapy service site. They help improve the quality of care offered, shape positive service provision behavior, remove unwanted variation in care processes and provide a frame-work for measuring results [3].

In Uganda, the AIDS Control Program (ACP) adapted the World Health Organization (WHO) standards of quality HIV/AIDS care, based on the national norms and needs of the country [2]. Some of the WHO standards adapted to the Ugandan context include: i) Provision of cotrimoxazole prophylactic therapy for all people living with HIV; ii) Routine screening of active tuberculosis (TB) among people living with HIV; iii) Assessment of ART eligibility using WHO clinical eligibility criteria or CD4 cell count; iv) Provision of standard ART regimen for eligible HIV clients and v) ART adherence monitoring.

WHO recommends the use of Cotrimoxazole Prophylactic Therapy (CPT) for patients with symptomatic HIV infection [4]. A number of studies have also shown that Cotrimoxazole prophylactic therapy prevents the occurrence of fatal opportunistic infections among patients with HIV [5,6]. The Uganda national ART guidelines recommend CPT for all HIV infected adults and children regardless of whether they are on antiretroviral therapy or not [7].

The World Health Organization estimates that about 5-15% of people living with HIV (PLHIV) have active tuberculosis (TB) based on symptom screening. Hence WHO recommends that all PLHIV need to be evaluated for active TB disease through intensified case-finding at the time of initial HIV diagnosis and during follow-up [4]. Evidence has also shown that intensified case-finding and treatment of TB among HIV infected persons interrupts disease transmission [8-10]. In light of this, the Ugandan Ministry of Health introduced intensified TB case finding tools at all care entry points, including the HIV/ART clinic to screen for active TB among HIV clients receiving care and there is provision to monitor the TB status of all HIV clients in the patient records. However, uptake of intensified case finding of TB is hampered by poor staff attitude towards the added documentation and ensuring that all the presumed TB cases receive appropriate laboratory tests.

Another key standard for ART in Uganda is assessment of ART eligibility using WHO clinical eligibility criteria or CD4 cell count. According to the Ugandan ART guidelines, the decision to initiate ART is based on the WHO clinical staging and CD4. WHO clinical staging criteria are a set of signs and symptoms of opportunistic infections or disease that affect people living with HIV. They manifest and are graded in severity from stage I - IV with declining immune status of the patient. However, in ideal situations, a CD4 count is essential for ART initiation and subsequent monitoring of the patients [2].

In Uganda, despite rigorous criteria for determining HIV clients eligible for ART, not all HIV clients in need of ART have access to treatment. It was for instance estimated that, 230,000 (42%) out of the 550,000 patients in need of ART accessed the services by end of 2010 [11]. The low uptake of ART is attributed to various challenges including inadequate skilled staff, high workload and lack of motivation among providers, lack of equipment/supplies, drug stock outs and inadequate systems to ensure that eligible patients are initiated and retained on ART [11]. Less than half (49%) of recommended medical staffing positions at hospital level in Uganda are filled [12]. There is also limited capacity at the regional and district level to conduct adequate support supervision and ensure providers adhere to recommended standards of quality HIV/AIDS care.

A possible consequence of rapid ART scale up in a weak health system is failure to adhere to standards of HIV/AIDS care leading to poor quality services. However, there is a dearth of data on how well clinicians in Uganda adhere to HIV/AIDS treatment guidelines. Hence the purpose of the study was to assess adherence of health facilities to standards of quality HIV/AIDS care in the country.

## Methods

### Study setting

The study was conducted in the West Nile region, located in the north western part of Uganda. It comprises 8 districts and has a total population of about 1,800,000 people who are predominantly subsistence farmers. The region has a total fertility rate of 7.2 per woman, which is slightly higher than the national average of 6.9 [13].

There are a total of 199 health facilities including a Regional referral hospital; General hospitals (9); Health Center IV’s (7); Health Center III’s (80); and Health Center II’s (92). A total of 20 health facilities were accredited by the MOH to provide ART in West Nile in 2008 [14]. These included one regional referral hospital, 8 general level hospitals, 8 health centre IV’s and 3 health centre III’s. The Uganda MOH maintains an accreditation system for facilities providing ART so as to ensure adherence to performance standards and to improve the quality of services provided. Facilities are authorized to provide ART if they meet set guidelines/standards which include availability of trained staff; accessible HIV counseling and testing; functioning laboratory services; a health management information system and a functioning follow up and referral system [14]. The MOH ensures that the standards are observed and monitored through trainings, provision of management protocols and support supervision.

### Study design, sampling and data collection

This was a cross sectional study conducted in 9 health facilities that met the selection criteria: Active health facilities in West Nile region that are accredited by the MOH to provide ART and records of HIV clients who were started on ART at least 12 months prior to the study. Accredited health facilities that were not active at the time of the assessment or had provided ART for less than one year and records of HIV clients who transferred in to the facility less than 12 months before data collection were excluded.

Records of 270 clients from the 9 health facilities that met the selection criteria were examined. Each health facility contributed a minimum sample of 30 client records (30 × 9 health facilities) to make up the total sample size.

All the nine health facilities that met the selection criteria were purposively included in the sample. Records of the eligible HIV clients that met the selection criteria were obtained through systematic sampling. The sampling population (N) for each facility (cohort of HIV clients that started ART at least 12 months prior to the study i.e. in the months of April - June 2009 was determined). The sampling population was then divided by 30 (the required sample size for each health facility) to get the sampling interval. The first file to be reviewed was randomly selected by flipping through the row of files while the subsequent files were identified by skipping and picking the file after every sampling interval for that facility, until when the last file was reached. This made up for the required sample size of 30 per facility and 270 for all the 9 facilities surveyed. In facilities where less than 30 patients enrolled on ART in that quarter, records of HIV/AIDS clients who enrolled in the preceding quarter of (Jan-Mar 2009) were considered.

Standards of quality HIV/AIDS care and ART were measured using indicators that represent the rate of accomplishment of the standard or the expected threshold. These included:

1. Proportion of HIV positive clients on ART who were prescribed daily cotrimoxazole
2. Proportion of HIV positive clients on ART who were assessed for active TB during each clinical follow up visit
3. Proportion of HIV positive clients on ART who were assessed for ART eligibility (using WHO clinical stage or CD4 count), prior to ART initiation)
4. Proportion of HIV positive clients on ART who were prescribed a standard ART regimen at the start of treatment
5. Proportion of HIV positive clients on ART who were assessed for adherence to antiretroviral (ARV) medicines on every clinic visit
6. Availability of basic laboratory tests for monitoring toxicity and response to ART.
7. Availability of ARV drugs, TB drugs and other medicines for treating opportunistic infections (O.I)

Quantitative data on adherence to clinical standards of HIV/AIDS was collected using an HIV/ART care cohort review form , a tool adapted from WHO and validated for use by the MOH/ACP for conducting cohort analysis of HIV clients enrolled on ART after every 6 months on treatment. Data on availability of drugs and supplies as well as laboratory functionality was collected using a facility observation check list adapted from the MOH health unit bi-monthly medicines availability and order checklist developed by the national Medical Stores.

### Data analysis

Data collected was cross checked for completeness and accuracy before being entered into Epi info version 3.5.1 and exported to Microsoft Access for further re-coding. Analysis was carried out in SPSS version 16 for windows and involved the estimation of proportions. Basic information about health facilities was analyzed according to the name, district, ownership, level and location.

Indicators to measure the standards of quality HIV care were analyzed as proportions with the number of times the provider complied with the standard in the numerator and the total number of clinical sessions or opportunities for complying the standard in the denominator. The performance of each health facility on the different indicators of standards of care was determined and compared to the national benchmarks.

Permission to conduct the study was obtained from the Uganda National Council for Science and Technology after the Makerere University Higher Degrees Research and Ethics Committee granted ethical approval. Permission was also obtained from the District Health Office supervising the respective health facilities. Unique codes were used to identify individual patient records in order to ensure confidentiality.

### Results

Two thirds of the facilities were public institutions and at the level of a general hospital while over half (55.6%) of the facilities were located in rural settings (Table 1). Prior to ART initiation, 254 (94.1%) of the HIV clients at all the facilities, had been assessed for ART eligibility using WHO clinical staging and the difference by level of facility was statistically significant (χ^2^ = 100.59, P = <0.001) (Table 2).

**Table 1:** **Distribution of the facilities according to various characteristics (N = 9)**

**Characteristic**	**Frequency (n)**	**Percentage (%)**
**Ownership**		
Public	6	66.7
Faith Based/PNFP	3	33.3
**Level**		
Regional referral hospital	1	11.1
General hospital	6	66.7
Health Center	2	22.2
**Location**		
Rural	5	55.6
Urban	4	44.4
**ART Client load as at June 2010**		
1000 - 6000 clients	2	22.2
301 - 999 clients	3	33.3
100 - 300 clients	4	44.5

ART: Anti Retroviral Therapy.

PNFP: Private Not For profit.

**Table 2:** **Proportion of HIV clients with baseline standards observed**

**Characteristics**	**Regional hosp (n = 30)**	**General hosp (n = 180)**	**H/center (n = 60)**	**Total (N = 270)**	**χ** ^**2**^ (2*)	**P-value**
**WHO stage**	16 (53.3%)	178 (98.9%)	60 (100.0%)	254 (94.1%)	100.59	<0.001
**CD4 count**	29 (96.7%)	114 (63.3%)	32 (53.3%)	175 (64.8%)	17.0	< 0.001
**Basic lab tests**	23 (76.7%)	76 (42.2%)	22 (36.7%)	121 (44.8%)	14.4	0.001
**Prescribed preferred 1st line ARV regimen **(AZT/3TC/NVP or EFV)	28 (93.3%)	126 (70.0%)	32 (53.3%)	186 (68.9%)	15.24	<0.001

*degrees of freedom; AZT - Zidovudine; 3TC - Lamivudine; NVP - Nevirapine; EFV - Effervirenz.

On average about two thirds (64.8%) of the HIV clients obtained a baseline CD4 test. However, almost all (96.7%) of HIV clients enrolled in care at the regional referral hospital obtained a baseline CD4 test compared to only 53.3% of clients in the health centers and 63.6% of those in general level hospitals. Similarly, about three quarters (76.7%) of HIV clients in the regional referral hospital obtained basic laboratory work-up prior to initiating ART. There was a statistically significant difference in the proportion of HIV clients that obtained basic laboratory work-up prior to initiating ART by level of facility (χ^2^ = 14.4, P = 0.001). Ninety three percent of HIV clients at the regional referral hospital and only half (53.3%) of HIV clients at the health centers were prescribed the preferred 1st line ARV combination recommended by the MOH (Table 2). The general hospitals and the health centers still had a notable proportion (27.2% and 46.7%) respectively of HIV clients being initiated on an alternative 1st line ARV regimen, one no longer being promoted at facilities, except in special circumstances such as for children under 5 years and when the preferred ART regimen is not available.

**Figure 1:**
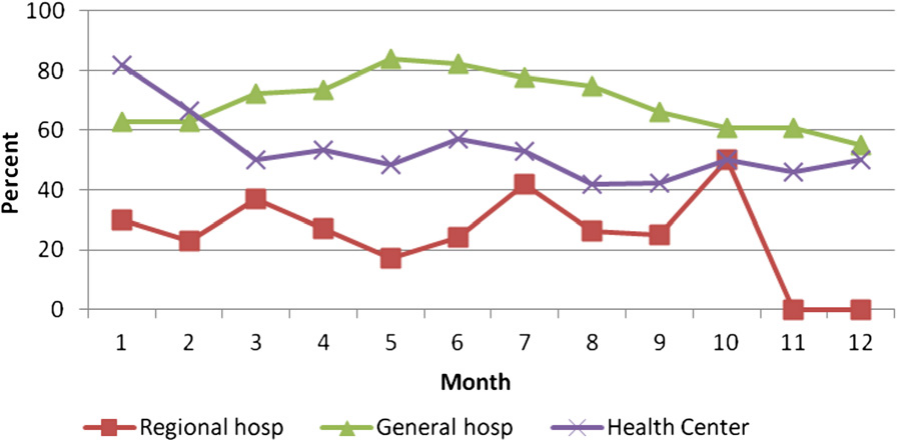
**Proportion of HIV clients on antiretroviral therapy assessed for adherence to treatment.** Hosp: Hospital.

Only 25% and on average 50% and 75% of the HIV clients on ART at the regional referral hospital, health centers and the district hospitals respectively, were assessed for adherence to ART during the follow up visits (Table 3). There was a general reduction in the proportion of HIV clients on ART that were assessed for adherence to treatment during follow up , from over 60% during initial visits at both general hospitals and health centers to about 50% over the 12 months while in care. The regional referral hospital improved over time up to tenth visit but still declined towards the last visits (Figure 1).

**Table 3:** **Percentage score per facility for each indicator of quality HIV/AIDS care**

**Standard/indicator**	**Arua RRH**	**Koboko H/C**	**Midigo H/C**	**Kuluva hosp**	**Maracha Hosp**	**Moyo hosp**	**Nebbi hosp**	**Nyapea hosp**	**Yumbe hosp**
**% of HIV clients in care who are prescribed daily cotrimoxazole**	100.0	99.3	98.5	98.5	99.7	93.8	94.4	97.2	97.1
**% of HIV clients in care who are assessed for active TB on every visit**	3.4	86.3	76.5	98.7	88.3	68.0	94.3	100.0	78.1
**% of HIV clients done WHO clinical stage at baseline**	53.3	100.0	30.0	100.0	96.7	100.0	96.7	100.0	100.0
**% of HIV clients done CD4 at baseline**	96.7	83.3	7.0	90.0	76.7	66.7	97.7	3.3	46.7
**% of HIV clients starting ART prescribed an appropriate ARV regimen**	100.0	100.0	100.0	100.0	100.0	100.0	100.0	100.0	100.0
**% of HIV clients on ART provided ART adherence counseling on every clinic visit**	25.0	50.0	56.0	88.0	99.0	59.0	80.0	72.0	49.0
**% of HIV clients eligible for ART who get laboratory work up before initiating ART**	76.7	66.7	6.7	76.7	10.0	56.7	76.7	13.3	20.0
**% of clients on ART who get at least one CD4 test result over a year**	36.7	10.0	3.3	6.7	16.7	10.0	16.7	0.0	3.3

RRH: Regional Referral Hospital.

TB: Tuberculosis.

WHO: World Health Organization.

CD4: Cluster of Differentiation for T-Lymphocyte.

ART: Anti Retroviral Therapy.

**Figure 2:**
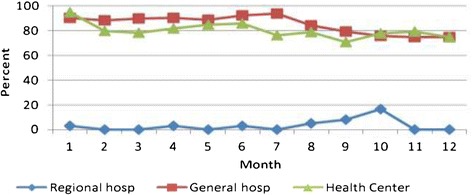
**Proporation of HIV clients on antiretroviral therapy assessed for active TB.** TB: *Tuberculosis.* Hosp: *Hospital.*

Only 3.4% of the HIV clients at the regional referral hospital had their TB status checked during each clinic visit. By contrast, an average of 88% of clients in both general hospitals and health centers had their TB status checked during each clinic visit (Table 3). The trends in performance at the facilities showed a general decline in observing the standard during the subsequent clinic visits, particularly at the general hospitals and the health centers, from over 90% during the initial 1–3 visits to about 75% in the subsequent visits (Figure 2).

## Discussion

We found that adherence to HIV/AIDS diagnostic and treatment guidelines were generally poor across all the health facilities assessed in Uganda’s West Nile region. Apart from the regional referral hospital which assessed clients for CD4 count, most health facilities still relied on the WHO clinical staging criteria to determine eligibility for starting ART. This practice is contrary to Uganda’s ART guidelines which clearly specify that the decision to initiate ART should be based on both the clinical staging and CD4 count. A CD4 count is essential for ART initiation and subsequent monitoring of the patients hence, anyone who is put on ART must have blood drawn for a baseline CD4 cell count within 3 months of initiation [14].

We also found that most of the facilities did not offer basic laboratory work up prior to ART initiation contrary to the national ART guidelines which recommend basic laboratory monitoring of HIV clients on ART for toxicity and treatment response of ART. Shortage of manpower and laboratory supplies was the major barriers to attaining the required standards of care. Failure to provide adequate laboratory monitoring to HIV clients initiating ART exposes patients to the risk of serious adverse ARV drugs reactions. It may also lead to delays in changing treatment regimen as treatment failure may not be detected early. Our findings are similar to those of an earlier study in Tanzania that found inadequate trained personnel and laboratory equipment as one of the reasons for poor quality ART services [15].

A combination of Zidovudine (AZT), Lamivudine (3TC) and Nevirapine (NVP) or Effervirenz (EFV) is the preferred first line Antiretroviral (ARV) regimen for Uganda. However, we found that a substantial proportion of the lower level facilities (health centers) were not initiating patients on the preferred first line ARV regimen. Stavudine (D4T) based ARV regimen was still being provided despite the fact that the MOH no longer recommends it because of the high risk of adverse effects especially peripheral neuropathy and lipo-dystrophy associated with long term use. Our findings are consistent with those of an earlier study in Uganda which found that more than 25% of ART facilities in the country were not observing the recommended standard for initiating HIV clients on ART [16]. The findings highlight the gap between policy and program implementation in the country.

We also found that basic standard of ART adherence monitoring and screening for active TB during each follow up visit was inadequate particularly at the regional referral hospital. In addition, the performance of the other facilities also declined with the subsequent follow up visits. Our findings mirror earlier studies in Cote d ‘Ivoire and Uganda which found that most facilities maintained good adherence to standards of quality HIV care only in the first visit with performance declining over time [17,18]. The low performance of facilities over time could be due to heavy patient load and provider complacence. A study in Uganda by Obua et al. also noted poor performance in ART adherence monitoring at facilities particularly with dual organizational programs i.e. Governmental/Non-Governmental Organization (NGO) [19]. It is imperative that these gaps be addressed as long term success of HIV/AIDS care and antiretroviral therapy requires observation of the required standards. Failure to do so may result in increased patient morbidity and mortality, and increased risk of developing antiretroviral drug resistance.

A limitation of our study is that we relied on records review to determine provider adherence to standards. Client clinical records may quite often be incomplete or have inaccurate information such that our findings paint a different picture of the actual situation. However, we believe that our findings reflect the Uganda HIV/AIDS ART treatment program especially in areas that are predominantly served by public health facilities given that, our findings are consistent with those of earlier studies in the country and elsewhere.

## Conclusion

We found that adherence to standards of HIV/AIDS care at facilities in the west Nile region was inadequate. Performance was better at the start of ART but declined during follow up visits. Higher level facilities were more likely to adhere to standards than lower health units, probably because they are well-equipped and adequately staffed. The findings have important implications for the HIV/AIDS management and control strategy in Uganda. As HIV/AIDS care is scaled up, program managers should ensure that accredited treatment centers are strongly supported to adhere to standards. There is a particular need to ensure that lower level facilities are also well equipped and adequately staffed in order to improve adherence to standards of care at these facilities.
